# Truncation of the Cytoplasmic Tail of Equine Infectious Anemia Virus Increases Virion Production by Improving Env Cleavage and Plasma Membrane Localization

**DOI:** 10.1128/JVI.01087-21

**Published:** 2021-11-09

**Authors:** Xue-Feng Wang, Yu-Hong Wang, Bowen Bai, Mengmeng Zhang, Jie Chen, Xiangmin Zhang, Min Gao, Xiaojun Wang

**Affiliations:** a State Key Laboratory of Veterinary Biotechnology, Harbin Veterinary Research Institute of Chinese Academy of Agricultural Sciences, Harbin, China; b Department of Geriatrics and Gerontology, First Affiliated Hospital of Harbin Medical University, Harbin, China; Icahn School of Medicine at Mount Sinai

**Keywords:** EIAV, Env, lentivirus, cytoplasmic tail, cleavage, plasma membrane, HIV-1

## Abstract

Envelope glycoproteins (Envs) of lentiviruses harbor unusually long cytoplasmic tails (CTs). Natural CT truncations always occur *in vitro* and are accompanied by attenuated virulence, but their effects on viral replication have not been fully elucidated. The Env in equine infectious anemia virus (EIAV) harbors the longest CT in the lentiviral family, and a truncated CT was observed in a live attenuated vaccine. This study demonstrates that CT truncation significantly increased EIAV production, as determined by comparing the virion yields from EIAV infectious clones in the presence and absence of the CT. A significant increase in a cleaved product from the CT-truncated Env precursor, but not the full-length Env, was observed. We further confirmed that the presence of the CT inhibited the cleavage of the Env precursor and found that a functional domain located at the C terminus was responsible for this function. Moreover, CT-truncated Env was mainly localized at the plasma membrane (PM), while full-length Env was mainly localized in the cytoplasm. The CT truncation caused a dramatic reduction in the endocytosis of Env. These results suggest that the CT can modulate the processing and trafficking of EIAV Env and thus regulate EIAV replication.

**IMPORTANCE** The mature lentivirus envelope glycoprotein (Env) is composed of a surface unit (SU) and a transmembrane unit (TM), which are cleaved products of the Env precursor. After mature Env is heterodimerically formed from the cleavage of the Env precursor, it is trafficked to the plasma membrane (PM) for incorporation and virion assembly. Env harbors a long cytoplasmic tail (CT), which has been increasingly found to play multiple roles in the Env biological cycle. Here, we revealed for the first time that the CT of equine infectious anemia virus (EIAV) Env inhibits cleavage of the Env precursor. Simultaneously, the CT promoted Env endocytosis, resulting in weakened Env localization at the PM. We also validated that the CT could significantly decrease EIAV production. These findings suggest that the CT regulates the processing and trafficking of EIAV Env to balance virion production.

## INTRODUCTION

Envelope glycoproteins (Envs) of lentiviruses are viral structure proteins and are well known to play essential roles in the infectivity, tropism, and immunogenicity of lentiviruses (1). From a subcellular perspective, the biological cycle of Env is initiated by Env precursor synthesis in the rough endoplasmic reticulum (RER) and cleavage in the Golgi apparatus (2). Cleavage leads to the splitting of the Env precursor into two products, namely, the surface protein (SU) (e.g., gp120 in human immunodeficiency virus [HIV-1] and gp90 in equine infectious anemia virus [EIAV]) and the transmembrane protein (TM) (e.g., gp41 in HIV and gp45 in EIAV). The two cleaved products noncovalently bind to a heterotrimeric Env complex to form mature Env. Mature Env in the cytoplasm is then delivered to the plasma membrane (PM), where a majority of mature Env proteins are rapidly internalized via clathrin-mediated endocytosis, whereas a minority of mature Env proteins are incorporated with Gag to assemble virions (3–5). Therefore, subcellular recycling of mature Env is pronounced due to cytoplasmic transportation from the cytoplasm to the PM. Although Env has been studied as the top target for vaccine development in HIV-1 (6, 7), its molecular modulation at the subcellular level remains understudied, resulting in numerous controversial findings (8–11).

Within the Env protein, the structure of TM is relatively conserved compared to that of SU. Three subdomains exist in the lentiviral TM: the ectodomain, the membrane-spanning domain (MSD), and the C-terminal cytoplasmic tail (CT). Notably, the TM of lentiviruses harbors a very long CT compared to that of other retroviruses (12–15). In EIAV, the CT is much longer (∼200 amino acids [aa]) than those in HIV and simian immunodeficiency virus (SIV) (16). Numerous findings have indicated that the CT plays multiple roles in modulating Env (17, 18), including Env trafficking (19, 20), Env internalization (21, 22), and Env-Gag incorporation (23–25), in HIV-1 and SIV. Moreover, CT-truncated mutants of Env were obtained by generating a premature stop codon in HIV-1 (26, 27), SIV (28, 29), and EIAV (30). These CT-truncated lentiviruses were found to have reduced viral replication and pathogenicity *in vivo* (31, 32), but the detailed molecular mechanisms of these phenomena remain poorly understood. Taken together, these findings suggest that a common molecular mechanism may exist regarding the CT of Env for the modulation of lentivirus replication and virulence.

It is well acknowledged that lentiviral members, including HIV-1 and EIAV, share many similarities, including their genomic structures, morphologies, methods for molecularly regulating viral replication, and life cycles. We previously observed a G2130A mutation in the *env* gene of donkey dermal fibroblast-adapted EIAV (EIAV_FDDV13_), which generated the premature stop codon TGA, resulting in a 154-residue deletion at the C terminus of CT (33–35). The mutation significantly reduced the replicative capability of EIAV in equine monocyte-derived macrophages (eMDMs), which are natural target cells of EIAV (35). To explore the roles of the CT in the Env of EIAV, we herein report that a CT-truncated EIAV showed significantly increased virion production, further comparing the expression characteristics and subcellular levels of localization of CT-truncated and full-length Env. Our findings showed that the CT functioned as a negative-feedback regulator that inhibited the cleavage of the Env precursor and as a prohibitor of Env PM localization, thereby regulating EIAV virion production.

## RESULTS

### CT-truncated mutation of EIAV Env enhances virion yield.

**FIG 1:**
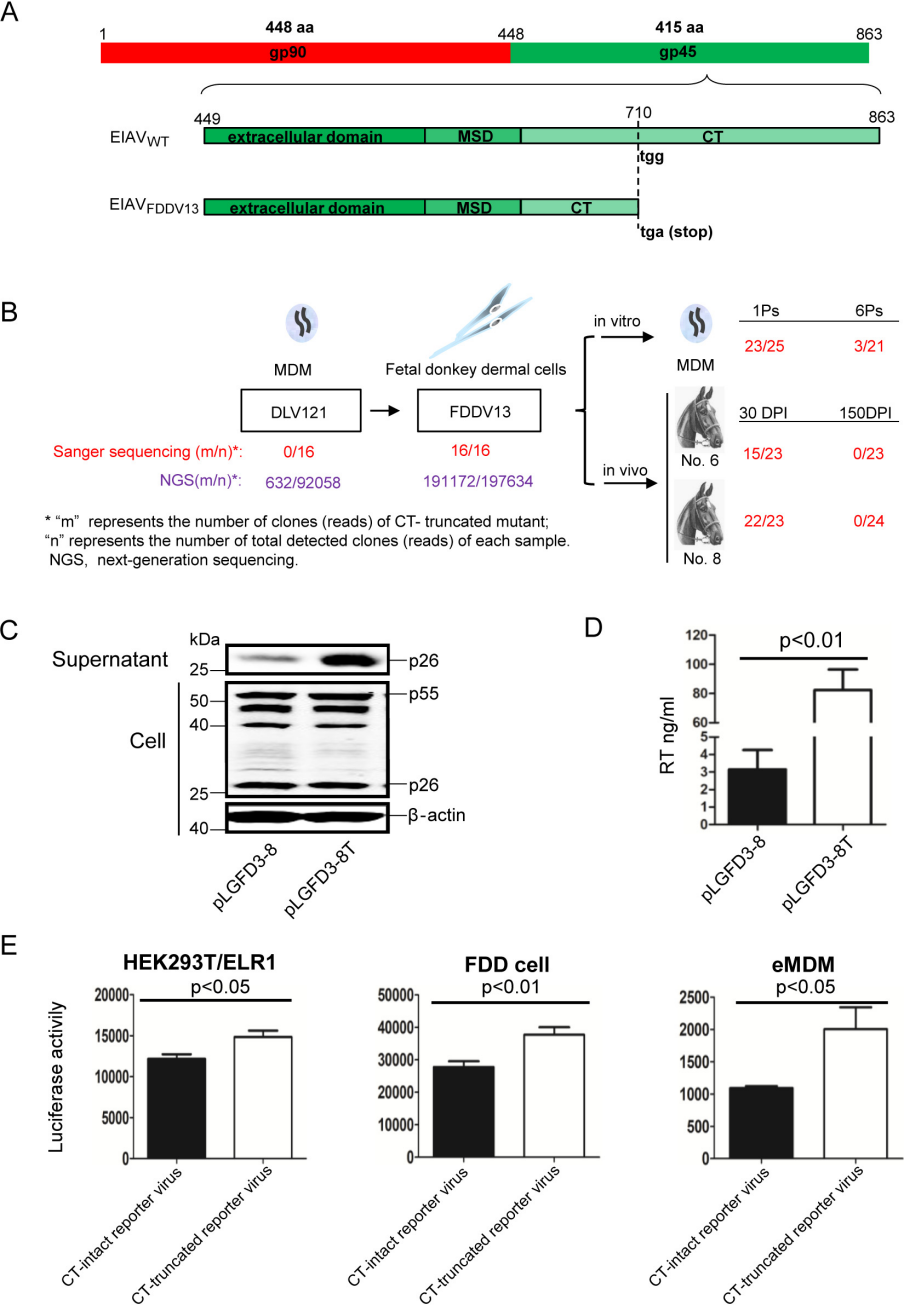
CT-truncated EIAV markedly increased the release of virions into the supernatant compared with CT-intact EIAV. (A) Schematic diagram depicting the structure of the truncated cytoplasmic tail (CT) on the Env precursor. The Env precursor is cleaved into gp90 and gp45. gp45 can be subdivided into three major domains, namely, the extracellular domain, membrane-spanning domain (MSD), and C-terminal CT. A premature stop codon (TGA) was produced in gp45 in fetal donkey dermal cell-adapted EIAV (EIAV_FDDV13_), which generated a 154-residue truncation in the CT, called the CT truncation mutation. EIAV_WT_, wild-type EIAV. (B) Characteristics of CT-truncated mutations. The gp45 sequences of EIAV_DLV121_ (a monocyte-derived macrophage [MDM]-adapted EIAV strain) and EIAV_FDDV13_ (13th generation EIAV_DLV121_ virus passaged in FDD cells) were sequenced by Sanger sequencing and next-generation sequencing (NGS). The gp45 sequences of EIAV_FDDV13_ from repassaging in equine MDMs *in vitro* and postinoculation of horses *in vivo* were sequenced by Sanger sequencing. The frequency of CT-truncated mutation was analyzed. Ps, passage; DPI, days postinfection. (C) CT-truncated mutation of Env enhanced virion yield. CT-truncated (pLGFD3-8T) or CT-intact (pLGFD3-8) EIAV infectious clones were transfected into HEK293T cells. The intracellular viral protein (Gag) and released virions (p26) were measured by Western blotting using an anti-EIAV p26 monoclonal antibody. This experiment was performed three times, and a representative result is shown. (D) The CT-dependent disparity of released virions was further confirmed by a comparison of reverse transcriptase activity. The data represent the means ± standard error (SE) from three independent experiments. (E) CT truncation mutation of Env promoted virion entry into target cells. Luciferase reporter viruses with a CT-truncated Env or a full-length Env were used to infect HEK293T/ELR1 cells, eMDMs, and FDD cells. The same amount (reverse transcriptase [RT]) of each reporter virus was inoculated at 24 h, the cells were lysed, and the luciferase activity of each reporter virus was examined. The data represent the means ± SE from three independent experiments.

Our previous study found that the *env* gene of EIAV_FDDV13_ had a high frequency of a premature stop codon induced by a G-to-A mutation at nucleotide 2130, truncating 154 aa (residues 710 to 863) in the C terminus of Env (Fig. 1A)—i.e., CT-truncated mutation of Env (33, 35). This study further investigated the occurrence of the mutation site and found an interesting phenomenon in which the CT-truncated mutation of Env exhibited almost 100% frequency in fetal donkey dermal (FDD) cell-adapted strain EIAV_FDDV13_, and this phenomenon was observed not even once in the macrophage-adapted parent strain, EIAV_DLV121_ (Fig. 1B). Moreover, reversion of the CT-truncated mutant of EIAV_FDDV13_ to a full-length phenotype was observed after passage *in vitro* in eMDMs and *in vivo* in infected horses (Fig. 1B), indicating that the CT-truncated mutation of Env may be an adaptive modulation in response to the corresponding microenvironment.

Previous studies have shown that the CT-truncated mutation of Env influences the replication capability of EIAV *in vitro* (35). To explore how the CT of Env affects EIAV replication, equivalent amounts of a CT-intact EIAV infectious clone (pLGFD3-8) and a CT-truncated EIAV infectious clone (pLGFD3-8T) were transfected into human embryonic kidney 293T (HEK293T) cells. The amounts of EIAV in the cell lysate and culture supernatant were measured as the levels of Gag (p55) and capsid (p26) by Western blotting. We managed to directly detect the expression of Env; however, Env was undetectable in both the cells and supernatant, partly due to the low sensitivity of EIAV-positive horse serum. A comparison of the two CT-distinctive EIAVs is shown in Fig. 1C. Despite no cellular difference in Gag, virions (p26) were released into the supernatant from cells transfected with the CT-truncated infectious clone at remarkably higher numbers than from cells transfected with the full-length CT infectious clones. This difference was further confirmed by reverse transcriptase (RT) activity (Fig. 1D). These results indicate that CT truncation is advantageous for EIAV production without affecting the synthesis of Gag, suggesting that the CT may regulate EIAV production.

Furthermore, a luciferase reporter pseudovirus harboring Env with or without a CT was simultaneously used to infected three target cells. Using luciferase activity as a proxy for virion entry, the quantities of viruses entering each target cell between the CT-truncated virus and the full-length CT virus were compared (Fig. 1E). The reporter activity of CT-truncated pseudo-EIAV was slightly increased compared to that of full-length CT pseudo-EIAV in all three target cells, indicating that the CT truncation mutation does not inhibit the virion entering into a target cell but rather has a weak facilitation effect.

### CT deletion enhances Env complex release and cleavage of the Env precursor.

**FIG 2:**
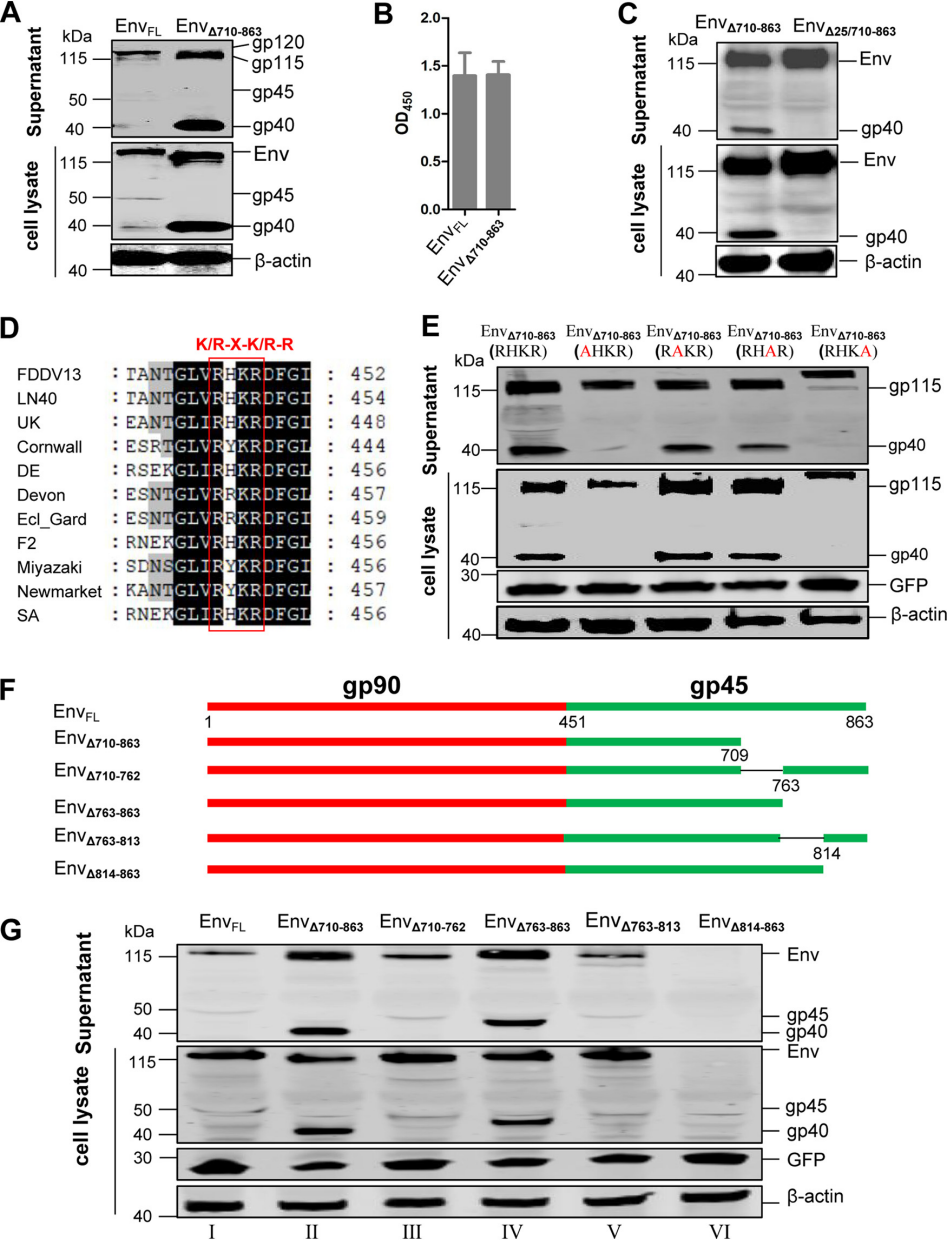
The CT regulates Env complex release and cleavage of the Env precursor. (A) Comparison of the expression levels of full-length Env (Env_FL_) and CT-truncated Env (Env_Δ710-863_). HEK293T cells were transfected plasmids expressing either the codon-optimized full-length or CT-truncated Env with HA tags. At 48 hpt, the cells were lysed, and the Env proteins in the culture supernatant were precipitated by ultracentrifugation. The Env proteins in the transfected cells and released into the culture supernatant were analyzed by Western blotting using an anti-HA monoclonal antibody. (B) The proliferative activity of HEK293T cells transfected with full-length or truncated Env expression vector was analyzed by CCK-8 assay. The data represent the means ± SE from three independent experiments. (C) Deletion of the 25 amino acids (aa 440 to 464) in the SU and TM adjacent regions in Env_Δ710-863_ changed its expression pattern and did not affect its release. HEK293T cells were transfected with plasmids expressing either Env_Δ710-863_ or Env_Δ440-464/710-86325_. The Env proteins in the transfected cells and released into the culture supernatant were analyzed by Western blotting. (D) Amino acid alignment of SU and TM adjacent regions of different strains of EIAV. The red box indicates the highly conserved motif (RX[H/Y/R]KR) in EIAV. The sequences are numbered according to the EIAV Env residues. (E) The RHKR motif is required for Env precursor cleavage. HEK293T cells were transfected plasmid expressing either the CT-truncated Env (Env_Δ710-863_ [RHKR]) or its mutant, as indicated. The Env proteins in the transfected cells and released into the culture supernatant were analyzed by Western blotting using an anti-HA antibody. GFP, derived from pEGFP-N1, was used as a transfection control. (F) Construction of different CT deletion mutations. The numbers represent the corresponding amino acid residues on the Env protein. (G) The constructs in panel F were transfected into HEK293T cells. Env proteins in the transfected cells and released in the culture supernatant were analyzed by Western blotting using an anti-HA antibody. Env_FL_ and Env_Δ710-863_ were used as negative and positive controls, respectively. GFP, derived from pEGFP-N1, was used as a transfection control. All experiments were performed three times, and a representative result is shown.

To further investigate whether CT modulates the synthesis and processing of Env, we constructed plasmids expressing full-length Env (Env_FL_ [gp120]) and CT-truncated Env (Env_Δ710-863_ [gp115]) with a hemagglutinin (HA) tag at the C terminus. After transfection into HEK293T cells, the amount of Env_Δ710-863_ released into the supernatant was remarkably increased compared to that of Env_FL_, although their expression levels were similar in the cell (Fig. 2A), and there was no difference in cell proliferative activity between HEK293T cells transfected with full-length Env and those transfected with the CT-truncated Env expression vector by CCK-8 assay (Fig. 2B). This result was consistent with that of Fig. 1C and indicated that CT deletion may increase Env and virion release. Interestingly, we detected a 40-kDa band (termed “gp40”) during the expression of Env_Δ710-863_ in both the cell lysate and the supernatant. In contrast, we hardly detected gp45 from Env_FL_ in the cell lysate or the supernatant (Fig. 2A). We hypothesize that gp40 is a “truncated version” of the cleaved product of gp45, which is cleaved from the CT-truncated version of the Env precursor Env_Δ710-863_.

To verify this hypothesis, we further deleted 25 amino acids (aa 440 to 464) in the SU and TM adjacent regions from Env_Δ710-863_, yielding Env_Δ25/710-863_. Intriguingly, we no longer detected gp40 in either the cell lysate or the supernatant (Fig. 2C). These phenomena suggest that gp40 was the cleaved product from Env_Δ710-863_ (i.e., CT-truncated gp45). It has been reported that the HIV-1 Env precursor is proteolytically cleaved by cellular furin or furin-like proteases (2, 36) and that the furin cleavage site is the highly conserved K/R-X-K/R-R-Y motif (X, any amino acid; Y, cleavage site) (37). By sequence alignment of the Env protein sequence of EIAV, conserved RX(H/Y/R)KR motifs were found in the SU and TM adjacent regions of EIAV Env (Fig. 2D). To confirm whether the RXKR motif affects the cleavage of Env, four Env mutants by alanine-scanning mutagenesis within the RHKR motif were constructed based on the CT-truncated Env and transfected into HEK293T cells (Fig. 2E). As expected, gp40 was observed in Env_Δ710-863_ (RHKR [a positive control]), Env_Δ710-863_ (RAKR), and Env_Δ710-863_ (RHAR), but not in Env_Δ710-863_ (AHKR) or Env_Δ710-863_ (RHKA). These results show that the cleavage of EIAV Env is dependent on the RHKR motif.

By comparing the levels of cleaved Env precursor in the presence or absence of the CT, we concluded that the CT may attenuate cleavage of the Env precursor. In addition, the increase in the expression of CT-truncated Env (Env_Δ710-863_ and Env_Δ25/710-863_) in the supernatant indicated that the CT may regulate the release of the Env complex. We also showed that the CT-dependent release of Env was independent of CT-modulated cleavage by deleting the cleavage site on Env (Fig. 2C and E).

### Identification of the potential functional domain on the CT for mediated release of the Env complex and cleavage of the Env precursor.

To identify which domain(s) of the CT functionally contributed to the regulation of Env complex release and cleavage of the Env precursor, we next constructed and expressed plasmids expressing four segment-deleted Envs (Env_Δ710-762_, Env_Δ763-863_, Env_Δ763-813_, and Env_Δ814-863_) based on Env_FL_ (Fig. 2F). To serve as negative and positive controls, both Env_FL_ and Env_Δ710-863_ were included. The expression of the remaining three segmentally CT-deleted Env proteins was compared with that of Env_FL_, except for Env_Δ814-863_, which expressed no protein in either the cell lysate or the culture supernatant (Fig. 2G, lane VI). The absence of Env protein in Env_Δ814-863_-transfected cells could not be due to reduced transfection efficiencies, since an equivalent level of green fluorescent protein (GFP) was expressed from the pEGFP-N1 plasmid, which was cotransfected as a control. In the culture supernatant, the expression of Env_Δ763-863_ was significantly increased (Fig. 2G, lane IV), while the expression of Env_Δ710-762_ and Env_Δ763-813_ was mildly increased (Fig. 2G, lanes III and V). However, in the cell lysate, compared to Env_FL_, none of the segmentally CT-deleted Envs showed remarkable changes in expression. Intriguingly, the supposed “truncated cleaved product” near the 40-kDa band was also detected when Env_Δ763-863_ was expressed. Therefore, these results indicate that some functional motifs between amino acids 763 and 863 of the CT may mediate inhibitory modulation upon Env precursor cleavage.

### The CT may inhibit cleavage of the Env precursor.

**FIG 3:**
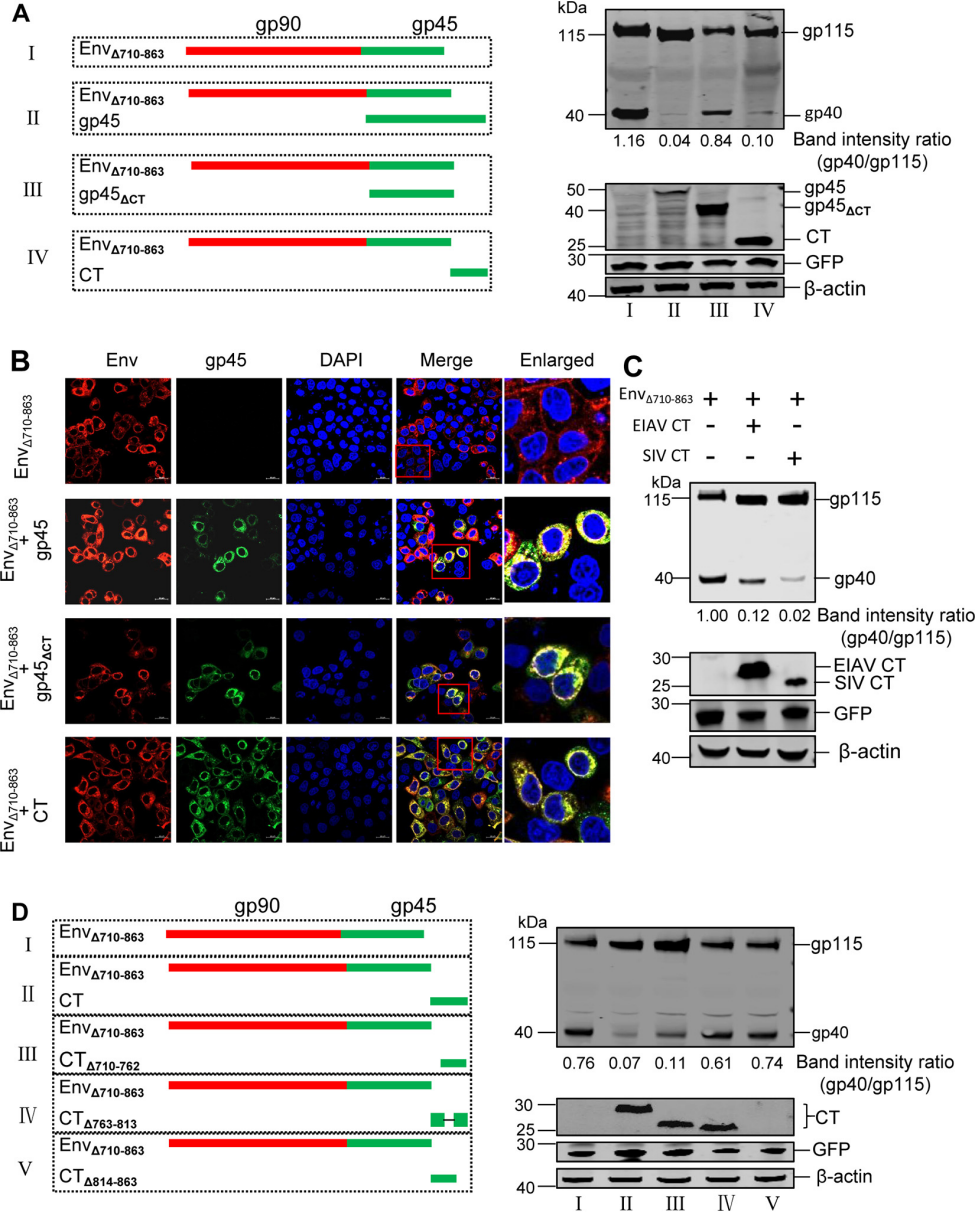
The CT can inhibit cleavage of the Env precursor. (A) The left panels schematically depict the coexpression among three Flag-labeled gp45 constructs (gp45, gp45_ΔCT_, and CT) and Env_Δ710-863_. A CT-truncated Env expression plasmid (Env_Δ710-863_) was transfected into HEK293T cells alone or with plasmids expressing Flag-tagged gp45 (gp45), Flag-tagged gp45_ΔCT_, or Flag-tagged CT. The cells were lysed, and the Env proteins in the cell lysate were analyzed by Western blotting. GFP, derived from pEGFP-N1, was used as a transfection control. The ratios of cleaved gp40 to uncleaved Env (gp115) in Western blots were quantified. (B) The colocalization of CT-truncated Env and gp45 or its variants was examined. HeLa cells were cotransfected with an HA-tagged vector expressing the CT-truncated Env (Env_Δ710-863_) and a Flag-tagged vector expressing gp45 or its variants. The cells were stained with an anti-HA antibody (red) and an anti-Flag antibody (green), nuclei were stained with DAPI (blue), and expression was analyzed by confocal microscopy. The red boxes highlight regions of images magnified in the enlarged panel. (C) SIV CT inhibited cleavage of the EIAV Env. The CT-truncated Env expression plasmid (Env_Δ710-863_) was transfected into HEK293T cells alone or with an expression plasmid encoding Flag-tagged EIAV CT or Flag-tagged SIV CT. The cells were lysed, and the Env or CT proteins in the cell lysate were analyzed by Western blotting. GFP, derived from pEGFP-N1, was used as transfection control. The ratios of cleaved gp40 to uncleaved Env (gp115) in Western blots were quantified. (D) The left panels schematically depict the coexpression of three Flag-labeled one-third-deleted CT constructs (CT_Δ710-762_, CT_Δ763-813_, or CT_Δ814-863_) and Env_ΔCT_. A CT-truncated Env expression plasmid (Env_Δ710-863_) was transfected into HEK293T cells alone or with an expression plasmid encoding Flag-tagged CT (CT), Flag-tagged CT_Δ710-762_, Flag-tagged CT_Δ763-813_, or Flag-tagged CT_Δ814-863_. The cells were lysed, and the proteins in the cell lysate were analyzed by Western blotting. GFP was used as a transfection control. The ratios of cleaved gp40 to uncleaved Env (gp115) in the Western blot were quantified. All experiments were performed three times, and a representative result is shown.

To further confirm the CT-dependent modulation of Env precursor cleavage, we constructed three plasmids expressing Flag-tagged complete gp45 (gp45), CT-truncated gp45 (gp45_ΔCT_), and the CT *per se* for cellular cotransfection with Env_Δ710-863_-expressing plasmids (Fig. 3A). The presence of the CT, either the CT on gp45 or the CT *per se*, could inhibit cleavage of the Env precursor, resulting in a reduction in Env cleavage efficiency (the ratio of gp40 to gp115) during the expression of Env_Δ710-863_ (Fig. 3A, lanes II and IV). However, the absence of the CT on gp45 abrogated the inhibition of Env cleavage, resulting in the presence of gp40 during the expression of Env_Δ710-863_ (Fig. 3A, lane III). These results validated that the CT plays an inhibitory role in Env precursor cleavage. The colocalization of these three Flag-tagged gp45 mutants with Env_Δ710-863_ was also examined. All of the gp45 mutants clearly colocalized with Env_Δ710-863_ (Fig. 3B), indicating that gp45 mutants and Env_Δ710-863_ can be expressed in the same subcellular compartment. In addition, we also detected that CT of SIV could inhibit Env_Δ710-863_ cleavage (Fig. 3C).

To further confirm the domain-dependent inhibition of CT modulation of Env precursor cleavage, we constructed three Flag-tagged CT deletion plasmids in which segments of the CT (one-third each) were deleted (CT_Δ710-762_, CT_Δ763-813_, and CT_Δ814-863_) for cellular cotransfection with Env_Δ710-863_-expressing plasmids (Fig. 3D). Cleavage of the Env precursor was reflected by the presence of gp40 during the expression of Env_Δ710-863_. We observed that cleavage was partially inhibited by the presence of CT_Δ710-762_ (Fig. 3D, lane III) but not CT_Δ763-813_ or CT_Δ814-863_ (Fig. 3D, lanes IV and V). Interestingly, the amino acids of CT_Δ710-762_ completely overlapped with the Env segment of aa 763 to 863, as described in Fig. 2D, suggesting that this region harbors a potentially functional motif (Fig. 2D). Therefore, these results indicated that the 100 aa from positions 763 to 863 may functionally regulate cleavage of the Env precursor.

### CT-truncated mutation enhances the distribution of Env at the PM.

**FIG 4:**
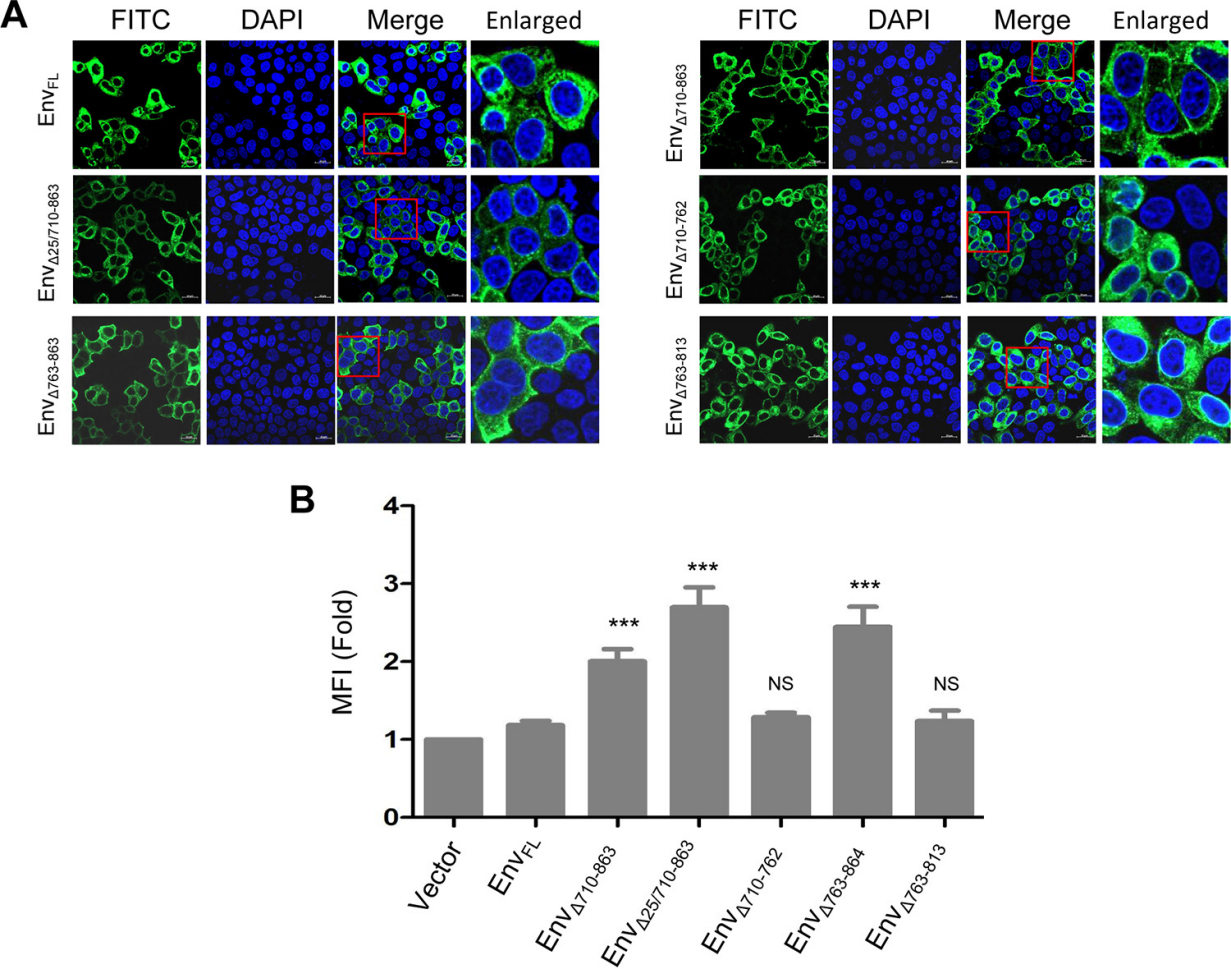
The CT can affect the subcellular localization of Env in cells. (A) HeLa cells were transfected with expression vectors encoding various Env proteins. The cells were stained with an anti-HA antibody (green), nuclei were stained with DAPI (blue), and the cells were analyzed by confocal microscopy. The red boxes highlight regions of images magnified in the enlarged panel. The red arrows indicate cytoplasmic localization, and the yellow arrows indicate plasma membrane (PM) localization. All of the experiments were performed three times, and a representative result is shown. (B) The PM expression of Env in HeLa cells transfected with expression vectors encoding various Env proteins was determined by flow cytometry using EIAV-positive horse serum, and the mean fluorescence intensities (MFIs) were compared to reflect the PM expression of Env protein in cells transfected with various Env protein variants. Compared with that of Env_FL_, the PM expression of Env_Δ710-863_, Env_Δ440-464/710-863_, or Env_Δ763-863_ was significantly increased (***, *P* < 0.01). The data represent the means ± SE from three independent experiments. NS, no significance.

Because the CT-truncated mutation increased the expression of Env in the supernatant, we further sought to determine whether the CT affects the subcellular localization of Env. The full-length Env expression plasmid and its CT truncation expression plasmid were transfected into HeLa cells (Fig. 4A). The majority of Env_FL_ was observed in the cytoplasm, while the majorities of Env_Δ710-863_ and Env_Δ25/710-863_ were observed at the PM. These results indicated that the CT-dependent subcellular localization of Env was independent of CT-modulated cleavage by deletion of the cleavage site on Env. To validate the potential function of the CT motif on the subcellular localization of Env, we visualized the subcellular localization of segment-deleted Env proteins. The expression of Env_Δ710-762_ was evenly distributed in the cytoplasm, the expression of Env_Δ763-863_ was largely localized at the PM, and the expression of Env_Δ763-814_ was largely observed in the cytoplasm. Moreover, we confirmed the CT-dependent subcellular localization on Env using flow cytometry (Fig. 4B). The subcellular expression patterns of segment-deleted Env proteins as determined by surface expression analysis via flow cytometry and subcellular visualization via confocal microscopy were highly similar. This CT segment-dependent disparity in subcellular localization further indicated that the CT, especially aa 763 to 863, may influence the subcellular localization of Env.

### CT may promote Env endocytosis.

**FIG 5:**
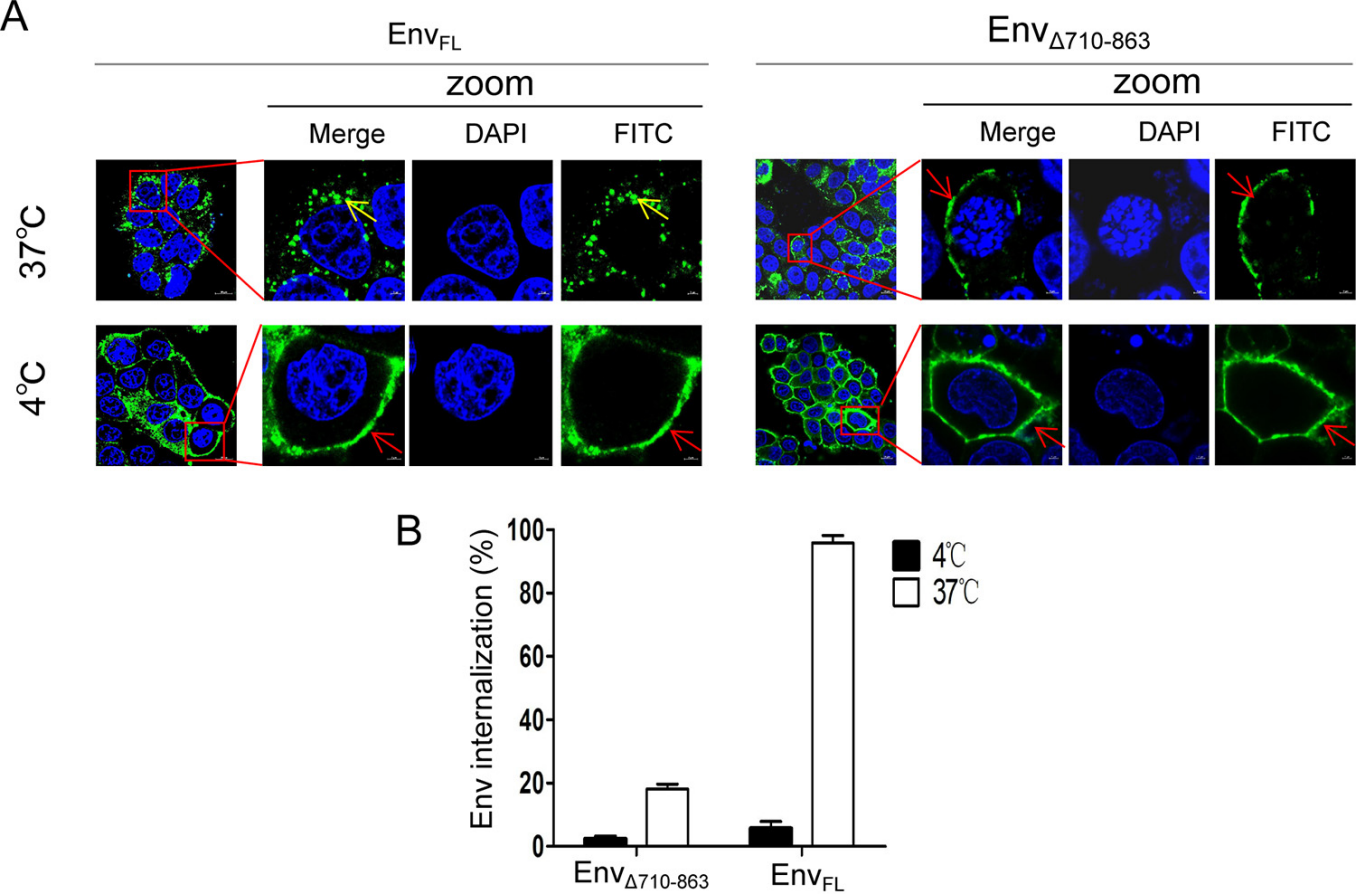
The CT can promote the internalization of Env in cells. (A) Using an antibody uptake assay, the levels of internalization of full-length Env and CT-truncated Env were compared. HeLa cells were transfected with plasmids expressing either full-length (Env_FL_) or CT-truncated Env (Env_Δ710-863_). The cells were incubated with EIAV-positive horse serum at 4°C for the essential antibody-antigen combination. Then, the cells were incubated at 37°C to detect intracellular internalization. The subcellular localization of Env_Δ710-863_ was unaltered, observed at the PM (red arrows) at both 4°C and 37°C. However, the subcellular localization of Env_FL_ changed from the PM (red arrows) at 4°C to the cytoplasm (yellow arrows) at 37°C, indicating observable internalization of Env. All experiments were performed three times, and a representative result is shown. (B) The levels of Env_FL_ and Env_Δ710-863_ internalization in panel A were calculated by the detected frequency of cells in which the Env protein was internalized from the PM. The figure represents 200 cells analyzed per sample from three independent experiments.

As shown previously, the CT of the primate lentivirus glycoprotein Env inhibits its cell surface expression by endocytosis (22), and we next investigated whether the CT of EIAV Env also regulates its subcellular distribution by endocytosis. An antibody-antigen uptake assay was performed. Using cells incubated at 4°C as a control for baseline internalization, the levels of internalization of Env_FL_ and Env_Δ710-863_ in cells incubated at 37°C were compared (Fig. 5A and B). When the temperature was increased from 4°C to 37°C, the intracellular localization of the majority of Env_FL_ changed from the PM to the cytoplasm (Fig. 5A and B), whereas the PM localization of Env_Δ710-863_ remained generally unchanged (Fig. 5A and B). This phenomenon indicated that the CT may promote the internalization of Env from the PM to the cytoplasm in cells.

## DISCUSSION

**FIG 6:**
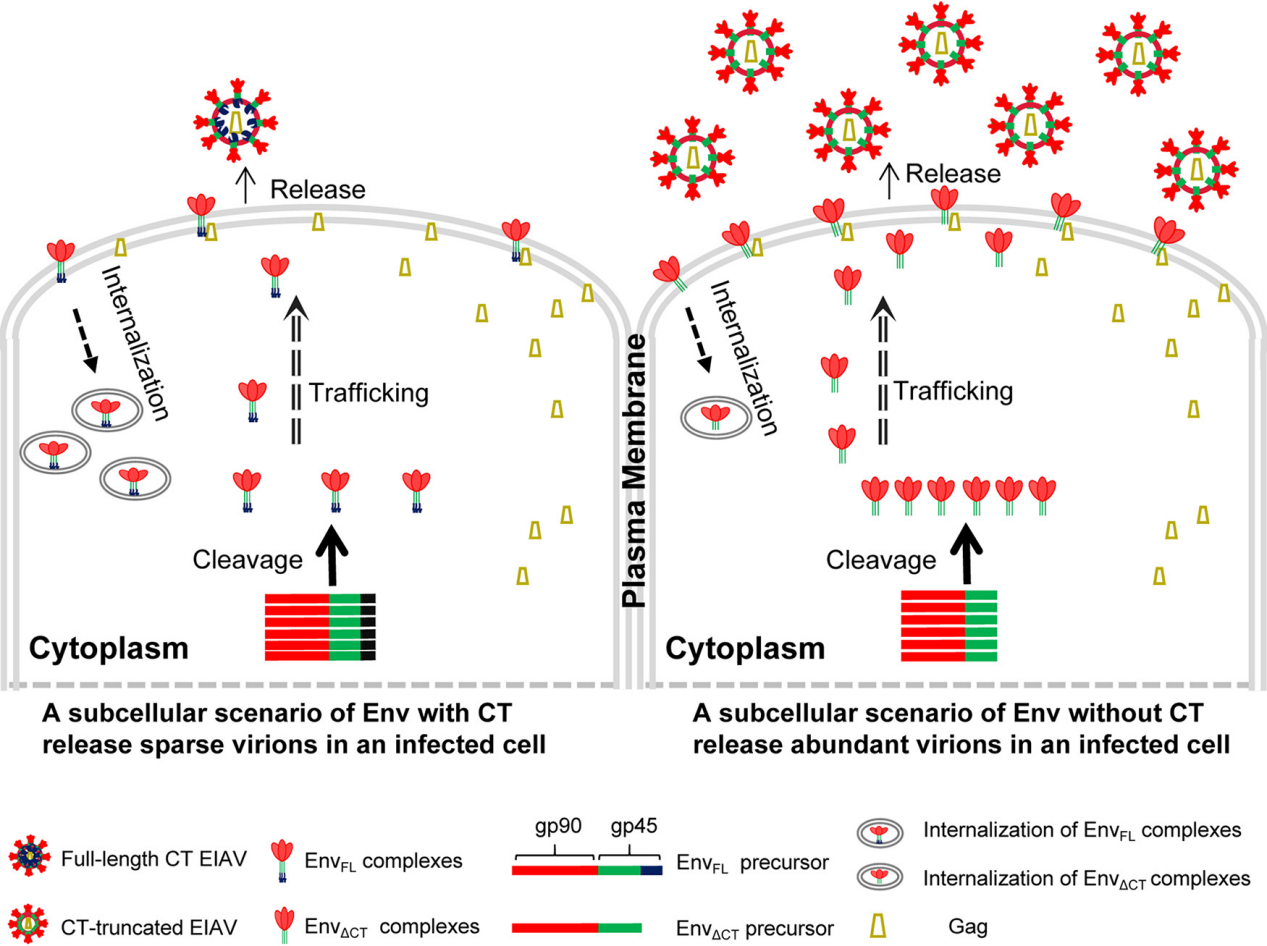
Schematic illustration of how the CT of Env affects the subcellular biological cycle of EIAV in an infected cell. The panel depicts the CT-dependent modulation of subcellular events regarding Env ranging from cleavage in the Golgi complex to localization at the PM. In the Golgi complex, the full-length Env precursor (left), consisting of gp90 (red bar) and gp45 (green bar), including the C-terminal tail (black ending), is cleaved into the surface subunit gp90 and the transmembrane subunit gp45 for immediate binding to the mature Env complex. The CT-truncated Env precursor (right), supposedly comprising gp90 (red bar) and CT-truncated gp45 (green bar), is cleaved into the surface subunit gp90 (red bar) and the transmembrane subunit (green bar) for immediate binding to the mature Env complex. The figure shows that the cleavage of the CT-truncated Env precursor was enhanced and/or accelerated, indicating that the CT may function as an inhibitor of Env cleavage in the Golgi complex. Internalization is accelerated in full-length Env compared with that in CT-truncated Env. Consequently, CT-truncated Env accumulates more at the PM and is released at greater levels into the extracellular matrix than full-length Env. These changes result in differential efficiency of viral assembly at the membrane. Finally, this demonstrates that the CT-dependent production of virions is thus attenuated in lentiviruses.

Numerous studies have shown that the CTs of primate lentiviruses, such as HIV-1 and SIV, have many functions related to the viral life cycle (13–15). However, EIAV has the longest CT, but few studies have investigated its biological functions. In this study, we found that a natural CT-truncated mutation of EIAV Env could significantly promote virion production and demonstrated that CT inhibited the cleavage of the Env precursor and decreased the levels of Env on the PM by accelerating Env internalization, suggesting that the CT impedes Env processing and transport and thereby decreases the EIAV virion yield (Fig. 6). The inhibition of Env expression by CT may contribute to low viral production and benefit viral escape from immune recognition and long-term infection.

One interesting phenomenon is that CT-truncated mutants of Env occur by generating a premature stop codon in SIV (28, 29) and EIAV (30), which is always accompanied by a decrease in virulence (31, 32). In this study, our data revealed that the CT truncation of EIAV occurred after passaging in FDD cells cultured *in vitro* and reverted to the full-length phenotype during replication in target cells or infected horses. A similar phenomenon has been observed in SIV (31). Lentiviruses, including EIAV, exist as complex quasispecies (38, 39), and the CT truncation is cell-type dependent, suggesting that the mutations are the result of an adaptive selection of viral quasispecies in different environments and may be accompanied by major compensatory changes in the involvement of host cell factors.

Cleavage of the HIV-1 Env precursor is required for viral infectivity (40–42) and is mediated by a cellular protease, furin (3). In this study, we identified the cleavage site of the EIAV Env precursor as the RXKR motif, which has the characteristics of a furin recognition sequence. Interestingly, CT of EIAV Env inhibits cleavage of the Env precursor and a functional domain located within 100 aa of the C terminus, which induces negative-feedback modulation. Accumulation of the cleaved product from the Env precursor triggers cleavage inhibition. However, the regulatory effects of the Env CT were too subtle to be detected in real time after Env_FL_ expression. Therefore, the Env cleavage effect was “amplified” in the absence of CT as an inhibitor, and subtle negative feedback of the CT on Env cleavage was observed. These results provide the first demonstration of the effects of CT modulations on the cleavage of lentivirus Envs. Because EIAV harbors the longest CT in the lentivirus family, this CT-dependent regulatory function in primate lentiviruses warrants confirmation, and the detailed molecular mechanism needs to be further studied. HIV-1 and SIV CT truncation mutations affect the structure of Env (17, 18, 43). Therefore, a truncation mutation of the CT might change the structure of EIAV Env and expose the cleavage site, which would further promote cleavage of the Env precursor. However, Sweety et al. showed that a deletion mutation of the CT in HIV-1 did not increase the efficiency of Env cleavage (17). Interestingly, our study found that cleavage of EIAV Env was inhibited by the presence of CT from SIV Env. Therefore, the main reason why CT inhibited Env cleavage may involve some host proteins, such as furin, which needs to be further studied.

A previous study showed that the release of CT-truncated Env of SIV was significantly increased compared with that of full-length Env, and a possible mechanism for this difference was that the CT-truncated Env primarily localized to the PM and was preferentially released from cells (44). We found that the released expression of truncated Env, such as Env_Δ710-863_ and Env_Δ763-863_, in supernatant was significantly higher than those of other Env variants, while there was no difference in their expression levels in cells. Such a CT-distinct disparity suggests that the CT might also modulate the subcellular localization of Env. Indeed, we found that CT truncation could increase the PM localization of Env in cells. Notably, similar subcellular localization patterns of CT-truncated Env have also been observed in HIV-1 (45) and SIV (10). Previous studies have found that a variety of host proteins interact with Env CT of HIV-1 or SIV and regulate the transport of envelope glycoproteins via the endocytosis pathway, thus affecting the expression of Env at the PM and virus production (19, 20, 46–48). Our data also indicated that the internalization of full-length Env was significantly stronger than that of CT-truncated Env, possibly because CT accelerates internalization of Env. Therefore, Env proteins without a CT could be easily released due to abundant PM localization, although potential host factors that may influence this process warrant further exploration. For example, Ying Liu et al. reported that the host protein P-selectin glycoprotein ligand 1 (PSGL-1) sequesters Env of HIV-1 at the PM by binding to gp41 (49). In addition, some studies have shown that Envs of HIV-1 release culture supernatant from HIV-1-infected cells or cells transfected with Env through the extracellular vesicle pathway (50, 51). Thus, another possible explanation for the increased release of truncated Env is that CT truncation facilitates Env incorporation in extracellular vesicles. Further work will be needed to elucidate this question.

Our study has two limitations. First, the present *in vitro* model merely examines viral factors in cells, and complicated host factors were not simultaneously studied. Second, very few monoclonal antibodies against EIAV could be commercially procured. We managed to overcome this issue with a series of CT-labeled plasmids and confirmed each subcellular event using at least two approaches. As the subcellular scenario involves both special and longitudinal biological events in infected cells, more systematic experiments are required.

The naturally truncated CT mutations were produced during the attenuation of the Chinese attenuated EIAV vaccine. We herein revealed that the existence of a CT-truncated mutation increased the efficiency of Env cleavage of the Env precursor, leading to promotion of expression of Env in the supernatant and enhanced virion production. It was documented that the cleaved Env of HIV-1 could more easily induce neutralizing antibodies (NAbs) than uncleaved Env (52, 53), indicating the CT might be a potential target for lentviral vaccine development. As such CT truncation was found in a widely used attenuated vaccine of the lentiviral family, our findings will provide an alternative strategy for novel HIV-1 vaccine development.

## MATERIALS AND METHODS

### Viral genome extraction and PCR.

EIAV gp45 sequences were amplified and cloned from proviral DNA. Genomic DNA was extracted using the Omega blood DNA kit (Omega, USA) from eMDMs infected with EIAV_FDDV13_ or from peripheral blood cells of EIAV_FDDV13_-infected horses (39). The complete Env sequence was amplified using primers p7-1 (TTG TAA GGT TTG GTG TAT GGG) and LTR-R (TGT TAG ATC TTG AAA ACA AGA C) with the genomic DNA from eMDM-infected samples as the template. PCR with 30 cycles of amplification was performed. The partial gp45 sequence was amplified using nested PCR primers, including the outer primers 45-1 (CCT CTG CAC CTA AGA TCC TCA GAG) and env-2 (CCG CTC GAG CTA AAC ATC ATA TTG AGG CAT TG) and the inner primers 45-a (CAT ACC TGG CAG ACG AGA CTC ATG) and 45-b (GCC CAT ATC CCA ACA AGC GTC CTA); the PCR amplification of *in vivo* samples was performed twice with 35 cycles per reaction. PCR was performed using TaKaRa LA *Taq* polymerase (TaKaRa, Dalian, China) according to the manufacturer’s instructions. All samples were subjected to three to five independent PCRs. After purification by agarose gel electrophoresis, the PCR products were ligated into the pMD18-T vector (TaKaRa, Dalian, China), and 15 to 25 clones from each sample were randomly selected for sequencing by Sanger methods.

### Cells and plasmids.

HEK293T and HeLa cells were cultured in Dulbecco’s modified Eagle’s medium (DMEM; Gibco, Thermo Fisher Scientific, USA) containing 10% fetal bovine serum (FBS). eMDMs were prepared from equine peripheral blood mononuclear cells (PBMCs) as previously described and were cultured in RPMI 1640 basic medium (GIBCO, Thermo Fisher Scientific, USA) containing 80% FBS. FDD cells were cultured in MEM Alpha Modification medium (HyClone, USA) containing 10% FBS (35).

CT-intact EIAV infectious clones (pLGFD3-8) were constructed based on EIAV_FDDV13_ as previously described (35). A CT-truncated EIAV infectious clone (pLGFD3-8T) was constructed based on pLGFD3-8. The clone was a mutant expressing an Env truncation of the gp45 gene by a G2130A substitution due to the presence of a stop codon. Using pLGFD3-8 or pLGFD3-8T as a template, expression plasmids for Env with or without a CT were amplified and inserted into the pcDNA3.1(+) mammalian expression vector (Invitrogen) by EcoRI and XhoI digestion, called pcDNAenv and pcDNAenvT, respectively. VRenv is a codon-optimized *env* gene-expressing plasmid that was kindly provided by Yiming Shao. Based on VRenv, a series of CT mutant plasmids, including HA-tagged, Flag-tagged, and segmentally deleted or partially deleted mutants, were constructed by PCR. Plasmid pcDNAEnv_FL_, which encodes a full-length EIAV Env, was constructed using the VRenv as the template, followed by subcloning into the pcDNA3.1(+) as a fusion with two HA tags at the C terminus. Plasmid pcDNAEnv_Δ710-863_, the CT-truncated Env expression plasmid, was constructed by deleting 154 amino acids at the C terminal of Env in the template plasmid pcDNAEnv_FL_. Plasmid pcDNAEnv_Δ25Δ710-863_ was constructed using the pcDNAEnv_Δ710-863_ as the template, which deleted 25 amino acids (aa 440 to 464) in the SU and TM adjacent regions. A set of deletion mutations of Env expression vectors, pcDNAEnv_Δ710-762_, pcDNAEnv_Δ763-864_, pcDNAEnv_Δ763-813_, and pcDNAEnv_Δ814-864_, were constructed by using pcDNAEnv_FL_ as the template. Plasmids pFlag-gp45, pFlag-gp45_ΔCT_, and pFlag-CT were constructed using the VRenv as the template, followed by subcloning into the pcDNA3.1(+) as a fusion with a Flag tag at the N terminus. The plasmid pFlag-CT was used as a template to generate the deletion mutant constructs pFlag-CT_Δ710-762_, pFlag-CT_Δ814-864_, and pFlag-CT_Δ763-813_. SIV CT was synthesized according to the SIV env sequence (GenBank accession no. M32741; nucleotide positions 2209 to 2646), cloned into pcDNA3.1(+), and fused to an N-terminal Flag tag. All constructed plasmids were verified by sequencing.

### Transfection and Western blotting.

HEK293T cells were transiently transfected with the indicated plasmids using calcium phosphate for 48 h. The culture supernatants were collected, and the cells were lysed in buffer containing 150 mM Tris-HCl (pH 7.6), 50 mM NaCl, 5 mM EDTA, and 0.1% Triton X-100. The culture supernatant was centrifuged at 12,000 × *g* for 10 min at 4°C to remove cell debris and centrifuged again at 20,000 × *g* for 2 h at 4°C to precipitate proteins. The proteins in the cell lysates and supernatant precipitates were separated by SDS-PAGE and transferred to polyvinylidene difluoride (PVDF) membranes (Millipore, Germany), which were blocked with 5% bovine serum albumin (BSA) in phosphate-buffered saline (PBS) for 2 h at room temperature. The membranes were incubated for 2 h with the appropriate primary antibodies. All Env proteins with HA tags were detected using a mouse monoclonal anti-HA antibody (Sigma, USA), and proteins with Flag tags were detected using a mouse monoclonal anti-Flag antibody followed by a secondary goat anti-mouse IRD800-conjugated monoclonal antibody (Sigma, USA). An antiactin polyclonal antibody was obtained from Sigma. All experiments were performed at least in triplicate.

### Immunofluorescence assay.

HeLa cells grown on polystyrene coverslips (Nest Biotechnology, China) were transfected with the various expression plasmids using PolyJet *in vitro* DNA transfection reagent (SignaGen, USA) according to the manufacturer's protocol. Cells were washed with PBS at 48 h posttransfection (hpt), followed by fixation in 4% (vol/vol) cold formaldehyde (Beyotime, China) for 15 min at room temperature. The cells were washed with PBS three times after fixation and then permeabilized with 0.1% (vol/vol) Triton X-100 for 15 min, followed by washing with PBS. The cells were blocked with 3% (wt/vol) BSA in PBS for 2 h and then labeled with a mouse (or rabbit) anti-HA antibody (Sigma, USA). The samples were then incubated with a goat anti-mouse IgG (whole molecule) fluorescein isothiocyanate (FITC)-conjugated antibody (Sigma) and a goat anti-rabbit IgG (H+L) highly cross-adsorbed secondary antibody conjugated to Alexa Fluor 633 (Thermo Scientific, USA) in PBS containing 3% BSA for 1 h at room temperature. Nuclei were stained with 2-(4-amidinophenyl)-6-indolecarbamidine dihydrochloride (DAPI) (Beyotime, China) for 15 min. Images were captured using a Leica DM-IRE2 confocal microscope (Leica, Germany). All experiments were performed in triplicate.

### Antibody-antigen uptake assay.

HeLa cells were transfected with pcDNAEnv or pcDNAEnv_Δ710-863_ for 24 h and then incubated with EIAV-positive horse serum at 4°C for 1 h. After complete removal of the antibody and washing 3 times with PBS, the cells were cultured with DMEM at 4°C or 37°C for an additional 30 min. The cells were then fixed with buffered 4% formaldehyde (Beyotime, China) and permeabilized with 0.1% Triton X-100. The cells were stained with a goat anti-horse IgGT H+L (FITC) secondary antibody (Abcam, USA). Images were captured using a Leica DM-IRE2 confocal microscope (Leica, Germany). All experiments were performed in triplicate.

### Flow cytometry.

HeLa cells were transfected with various HA-tagged Env-expressing plasmids for 48 h. After fixation with 4% paraformaldehyde, the cells were incubated with EIAV-positive horse sera for 1 h. After washing, the cells were incubated with a goat anti-horse IgGT H+L (FITC) secondary antibody (Abcam, USA) for 1 h. The mean fluorescence intensity of Env expression on the cell surface was determined by flow cytometry.

### Construction of and infection with a luciferase-expressing EIAV reporter virus.

A luciferase-expressing EIAV reporter virus was constructed by modifying a three-plasmid EIAV transfection system (54). HEK293T cells were cotransfected with pONY8.1-LUC and pEIAV-GagPol and pcDNAenv or pcDNAenvT. The reporter virus was collected 48 hpt, centrifuged at 1,000 rpm for 10 min to remove cell debris, filtered through a 0.45-μm-pore filter unit, and stored at –80°C. The luciferase reporter virus was quantified using a reverse transcriptase (RT) activity kit (reverse transcriptase assay, colorimetric kit; Roche, Switzerland) and used to infect HEK293T/ELR1 cells (an equine lentivirus receptor 1 [ELR1]-expressing HEK293T cell line) (54), FDD cells, and eMDMs in 96-well plates for 48 h. These seeded cells were washed and subjected to luciferase analysis.

### Statistical analysis.

Statistical analysis was conducted using GraphPad Prism, version 5 (GraphPad Software, USA), and Microsoft Excel (Microsoft, 2010, USA). Student's *t* test was used to compare the differences between the experimental and control groups.
